# Evaluation of a Continuous Indicator for Syndromic Surveillance through Simulation. Application to Vector Borne Disease Emergence Detection in Cattle Using Milk Yield

**DOI:** 10.1371/journal.pone.0073726

**Published:** 2013-09-12

**Authors:** Aurélien Madouasse, Alexis Marceau, Anne Lehébel, Henriëtte Brouwer-Middelesch, Gerdien van Schaik, Yves Van der Stede, Christine Fourichon

**Affiliations:** 1 INRA, UMR1300 Biologie, Epidémiologie et Analyse de Risque en santé animale, BP 40706, Nantes, France; 2 LUNAM Université, Oniris, Ecole nationale vétérinaire, agroalimentaire et de l’alimentation Nantes Atlantique, UMR BioEpAR, Nantes, France; 3 GD Animal Health Service, Deventer, the Netherlands; 4 Unit for Co-ordination of Veterinary Diagnostics, Epidemiology and Risk Analysis (CVD-ERA), Brussels, Belgium; 5 Department of Virology, Parasitology and Immunology, Faculty of Veterinary Medicine, Ghent University, Merelbeke, Belgium; Cornell University, United States of America

## Abstract

Two vector borne diseases, caused by the Bluetongue and Schmallenberg viruses respectively, have emerged in the European ruminant populations since 2006. Several diseases are transmitted by the same vectors and could emerge in the future. Syndromic surveillance, which consists in the routine monitoring of indicators for the detection of adverse health events, may allow an early detection. Milk yield is routinely measured in a large proportion of dairy herds and could be incorporated as an indicator in a surveillance system. However, few studies have evaluated continuous indicators for syndromic surveillance. The aim of this study was to develop a framework for the quantification of both disease characteristics and model predictive abilities that are important for a continuous indicator to be sensitive, timely and specific for the detection of a vector-borne disease emergence. Emergences with a range of spread characteristics and effects on milk production were simulated. Milk yields collected monthly in 48 713 French dairy herds were used to simulate 576 disease emergence scenarios. First, the effect of disease characteristics on the sensitivity and timeliness of detection were assessed: Spatio-temporal clusters of low milk production were detected with a scan statistic using the difference between observed and simulated milk yields as input. In a second step, the system specificity was evaluated by running the scan statistic on the difference between observed and predicted milk yields, in the absence of simulated emergence. The timeliness of detection depended mostly on how easily the disease spread between and within herds. The time and location of the emergence or adding random noise to the simulated effects had a limited impact on the timeliness of detection. The main limitation of the system was the low specificity i.e. the high number of clusters detected from the difference between observed and predicted productions, in the absence of disease.

## Introduction

Syndromic surveillance consists in the routine monitoring of one or several indicators for the early detection of adverse health events. It is an active area of research with applications spanning from the routine detection of seasonal flu [1] to potential bioterrorism threats [2]. Surveillance is also of interest in veterinary epidemiology for the early detection of the emergence or re-emergence of diseases [3], [4]. Our motivating problem is the emergence of 2 vector borne diseases in the European ruminant populations since 2006. The bluetongue virus, which had been spreading with growing intensity in the south of Europe since 1998 [5], emerged in north-western Europe in August 2006. Before the end of that year it had spread to the Netherlands, Belgium, Germany, Luxemburg and France [6]. In the second half of 2011, episodes of fever, milk drop and diarrhoea in cows were reported in Germany and the Netherlands [7]. A previously unknown orthobunyavirus was identified and named Schmallenberg virus after the town from which originated the first sample in which the virus was found [8]. Both bluetongue and Schmallenberg viruses are transmitted by culicoides midges. With the emergence of 2 vector borne diseases only 5 years apart and their potential link to climate change [9], we need to stand prepared for the emergence of new diseases, some of which could be zoonotic. Syndromic surveillance systems for the early detection of animal diseases should be developed and implemented.

All methods used for syndromic surveillance detect temporal or spatio-temporal patterns of deviations between expected and observed values produced by abnormal events on one or several indicators [10]. Therefore, the performance of any detection method depends crucially on the characteristics of the deviations induced by the abnormal event as well as on the performance of the model used to predict the indicators’ expected values. Compared to surveillance of known diseases, the fact that the next disease to emerge is unknown represents a difficulty for the evaluation of the performance of a detection method. Sensitivity can only be calculated when the distribution of the monitored indicator in the infected population is known. This means that in order to calculate sensitivity we can only work with hypothesised disease characteristics. In order to assess the quality of the prediction, the 2 elements to consider are i) **bias**, whereby predicted indicator values are too high for several consecutive time-intervals or adjacent locations leading to an apparent cluster of low values and ii) **noise**, whereby the disease effects are diluted in the unexplained indicator values’ variability.

A source of data that has not yet been explored for prospective syndromic surveillance in cattle is milk recording data. Milk recording consists in the measurement of milk quantities and constituents from all the cows of a dairy herd on a regular, mostly monthly, basis. A drop in milk yield can be expected to be a non specific and precocious symptom associated with most diseases, as milk production represents a high metabolic demand for the dairy cow [11]. Therefore, milk recording data are potentially good candidates to be included in a disease surveillance system, and this should be evaluated. How to carry out this evaluation is however non trivial. Indeed, the next disease to emerge, how it will spread and the extent of its effects on the milk production of individual cows are all, by definition, unknown. Furthermore, most of the indicators used for cluster detection investigated are discrete, and there are few methods and applications using continuous indicators for prospective syndromic surveillance [12].

The aim of this study was to present a framework for the quantification of both disease characteristics and model predictive abilities that are important for a continuous indicator to be sensitive, timely and specific for the detection of a vector-borne disease emergence. This was undertaken by simulating the emergence of a range of diseases and their impact on milk production in French dairy cattle in 2006.

## Materials and Methods

### Data

Milk recording is the regular collection of milk production data from all lactating cows in a herd. It is usually performed at monthly intervals, on two consecutive milkings. The number of kg of milk given per cow per day (milk yield) is measured and a milk sample is taken to determine milk composition, for all the cows of a tested herd. Farmers participate in order to get individual cow performance data. These data are also used for the genetic evaluation of sires and cows. In France, they are centralised by the *Centre de Traitement de l’Information Génétique* (**CTIG**) for this purpose. Milk recording data collected in herds enrolled in the French milk recording scheme between 2003 and 2006 were extracted from the CTIG national database. Records that originated from only one milking (as opposed to the usual 2 consecutive milkings) or from herd-recording dates (**test-day**) with less than 10 cows recorded were excluded. In total, 48,713 herds were included. Farmers have some control over lactation length and this can be used to adjust the quantity of milk sold to external factors. In order to limit this source of variation, only recordings from cows between 5 and 305 days in lactation were used. The median number of cows recorded between 5 and 305 days in milk per test-day in these herds was 29. Herd location was available at the municipality level. Three levels of data are used in the remainder of this manuscript. Milk losses were simulated at the individual cow-recording level. Prediction of expected milk quantities were carried out on mean milk productions per test-day. Disease detection was undertaken at the municipality/week level.

### Expected Milk Production

In order to detect a deviation from an expected milk production, the expected milk production must be predicted. Expected test-day milk productions in 2006, including cows recorded between 5 and 305 days in lactation only, were predicted using herd specific historical data as follows. Mean test-day milk production per cow between 2003 and 2005 was modeled using a linear mixed model. The model specification was:
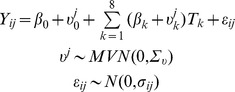
where 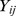 was the mean test-day milk production per cow recorded between 5 and 305 days in lactation on test-day 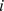 in herd 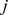, 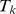 was the day of the year linearly interpolated on 2 out of 8 consecutive time intervals, 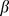 was a vector of coefficients associated with the effect of day of year on milk production, 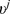 a matrix of herd random effects following a multivariate normal distribution with mean 0 and variance-covariance 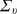 and 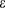 was a vector of residual error following a normal distribution with mean 0 and variance 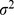. Parameters were estimated in R [13] using the lmer function [14]. Mean milk productions per cow were predicted for each test-day observed in 2006 using this equation with the parameters estimated between 2003 and 2005.

### Simulation of Disease Emergence

In order to determine the disease characteristics that are important for disease detection, a disease emergence model was developed. In a first step disease spread was simulated in space and time resulting in each cow being assigned a date of infection or the absence of infection. In a second step, for each observed recording date, a quantity of milk lost because of the disease was simulated.

**Figure 1:**
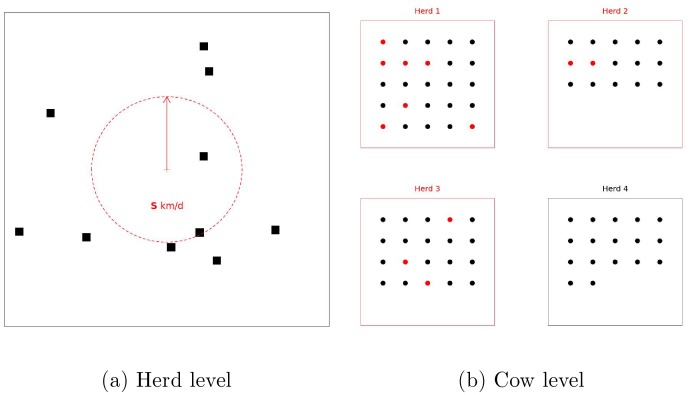
Model used for the simulation of disease spread. The disease spreads as a circle at 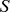 km/day. Once the disease has reached a municipality centroid, each uninfected herd in the municipality can become infected with a daily probability 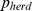. Once a herd is infected, each cow from the herd can become infected with a daily probability 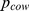. Squares on the left-hand side represent herds. On the right-hand side, black dots and red dots represent uninfected and infected cows respectively.

Disease spread was simulated as follows. From its starting location and date, the disease spread as a circle with speed 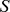 km/day. Once the disease had reached a municipality centroid, each herd from this municipality could get the disease with probability 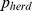 (Figure 1). Once a herd was reached by the disease, each cow from the herd could get the disease with probability 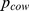 (Figure 1). Simulations were carried out with a daily time step, and, because we were interested in early detection, for a duration of 60 days after disease emergence. On each day of simulation, the statuses of each uninfected herd within an infected municipality and of each cow within an infected herd were sampled from a Bernoulli distribution with parameters 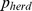 and 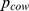 respectively. For each cow either the absence of infection or the day of the simulation at which the cow became infected were stored for the simulation of the effects of the disease on milk production.

**Figure 2:**
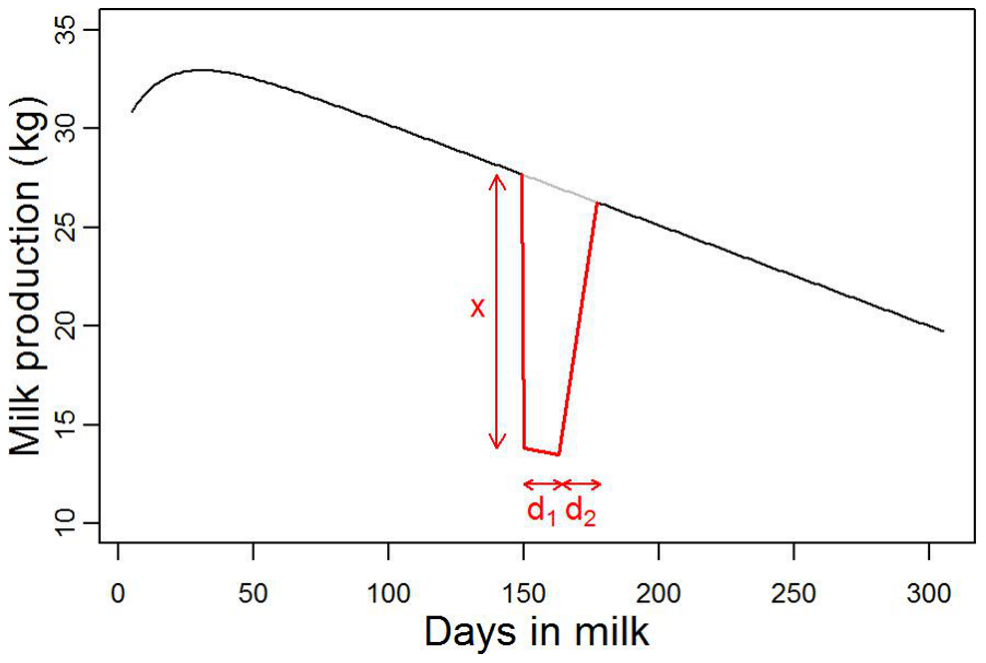
Simulation of milk losses caused by the disease represented on the theoretical lactation curve of a Holstein cow in her second lactation. Milk production drops by a proportion 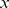 on the day of disease onset and for a period of 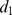 days. Milk production gets backs to its normal value linearly over a period of 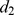 days. Milk losses were simulated on monthly recorded milk yields.

The effects of the emergences on milk production were simulated at the cow-recording level. The loss of milk production resulting from the infection was assumed to be proportional to the recorded milk yield and made dependent on the day since infection simulated at the previous step (Figure 2). More precisely, milk production was assumed to drop by 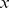 % on the day a cow became infected. The loss of milk production was maintained at this rate of 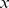 % for a period of 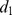 days. It then went back linearly to the ‘normal’ milk production over a period of 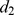 days.

**Figure 3:**
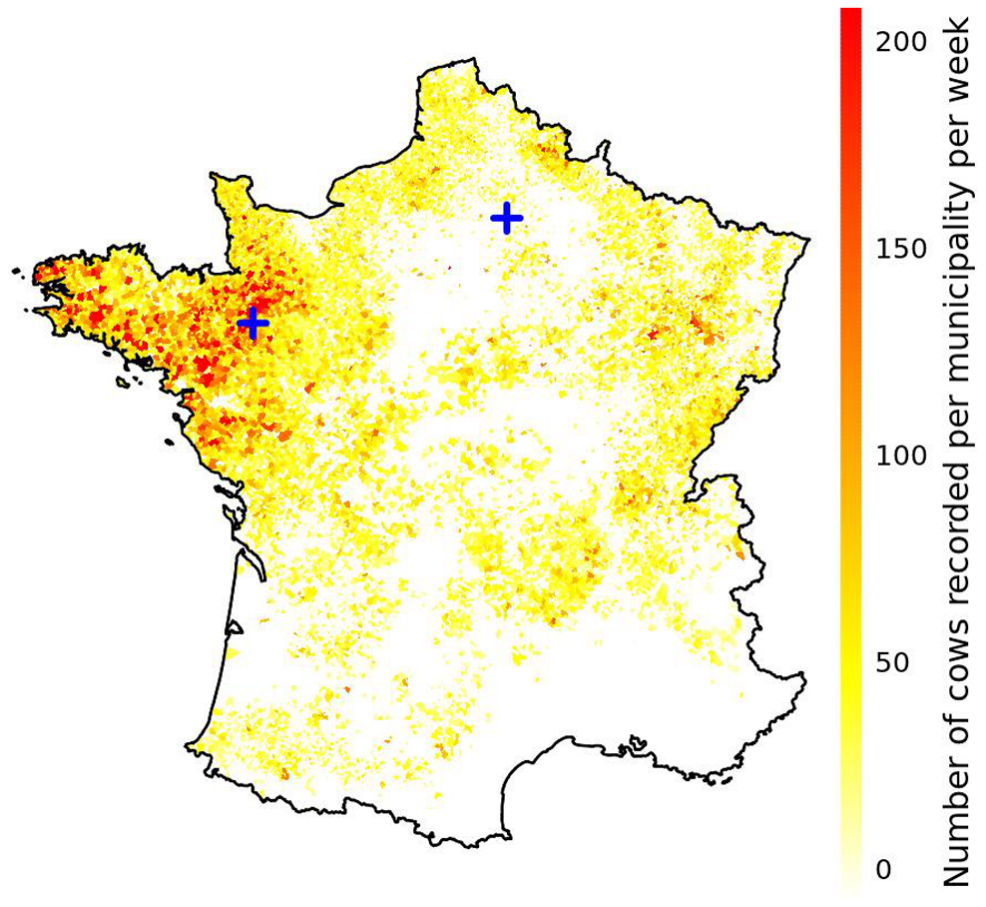
Mean number of cows recorded per municipality per week in the dataset used for simulation. The 2 blue crosses represent the disease starting locations used in the simulations.

The model was used to simulate a disease emergence in 2006. This year was chosen because only 6 bluetongue cases were reported in 2006 in France and the disease spread in a large number of herds in 2007. This could have had an effect on the milk production observed during 2007 and the following years. All the herds and cows recorded in 2006 were included in the simulations. The parameters used in simulations are presented in Tables 1 and 2. Two starting locations and 2 starting dates were used. One location, in the West, had a high cattle density and one, in the North, had a low cattle density (Figure 3). Diseases were made to emerge on the 1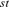 of March and the 1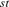 of September. Values selected for disease speed were chosen based on the published estimate of 5.6 km/day for the front wave velocity of bluetongue [15]. As there were no published values for the probabilities of infection within and between herds such as simulated here, a wide range of plausible values was used. Using the same values for 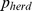 and 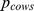 of 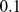 and 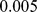, respectively 65% and 5% of the population would be infected 10 days after emergence, and, 99% and 26% would be infected 60 days after emergence. Three milk losses scenarios with different values for 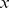, 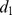 and 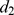 were run on each spread scenario and the resulting milk productions stored. Based on published estimates for the impact of bluetongue on milk production [16], the parameters were chosen so that the resulting loss of milk production would amount to around 3% of the milk produced between 5 and 305 days in lactation for a Holstein cow infected on day 150 of lactation. Simulations were run for all 192 combinations of spread parameters (Table 1) and 3 milk loss scenarios (Table 2) resulting a total of 576 simulated scenarios.

**Table 1:** Parameters used for disease spread simulation.

Parameter	Abbreviation	Values
Disease starting location		West, North
Disease starting date		01/03/2006, 01/09/2006
Disease speed	*S*	1, 5, 10 km/day
Daily probability of infection within municipality	*P_herd_*	0.005, 0.01, 0.05, 0.1
Daily probability of infection within herd	*P_cow_*	0.005, 0.01, 0.05, 0.1

See Figures 1 and 3 for details on the simulation model.

**Table 2:** Parameters used for the simulation of disease induced milk losses.

Parameter	Abbreviation	Sc 1	Sc 2	Sc 3
Proportion of recordedproduction lost at onset	*x*	0.9	0.5	0.35
Duration of loss (days)	*d_1_*	5	14	21
Duration back tonormal (days)	*d_2_*	14	14	21

Three combinations of parameters were simulated and combined with the disease spread simulations given in Table 1. See Figure 2 for details on the simulation model of milk losses.

### Space-time Patterns of Disease Induced Milk Losses

Simulated milk productions were generated for 576 scenarios representing all possible combinations of model parameters (See Tables 1 and 2). As it would have been impossible to attempt to detect disease emergences on all scenarios, groups of scenarios were created. The ability of a detection method which uses milk production to detect a disease emergence will depend on the spatio-temporal pattern of milk losses. To compare the scenarios based on this criterion, simulated milk losses were averaged per cow for 8 areas centered on the known disease starting locations, per week for 8 weeks after the emergence. The 8 areas were circles centered on the disease starting locations with radii of 20, 30, 40, 50, 100, 200, 500 and 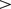 500 km. This gave for each scenario a grid of 64 values of milk production loss per cow per zone and per week after emergence. By design, milk losses between adjacent zones and between consecutive weeks were correlated. A principal component analysis was run on the 64 values characterizing each scenario in order to identify the combination of variables that allowed to discriminate best between the different scenarios. The k-means algorithm was then run on the identified principal components to make groups of scenarios with similar characteristics.

### Cluster Detection

The detection of a disease emergence based on its effects on milk production assumes that the emergence will result in a decrease in milk production that is localised in space and time. This type of deviation can be detected using a scan statistic [17]. Disease emergence detection was carried out with SaTScan™ using a space-time prospective analysis [18]. The cluster detection algorithm was parametrized assuming a normal distribution [12] for the indicator analysed. The model input was either the mean simulated losses per municipality per week or the mean difference between observed and predicted milk production per municipality per week (kg of milk/municipality/week) with each observation weighted by the square root of the number of cows recorded. In order to detect a drop in milk production on a given week, data for this week and the 4 preceding weeks were analyzed. This was done to feed the program with background information on milk production since milk recording is carried out on a monthly basis in most herds. Alarms were clusters with a p-value 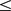 0.05. Cluster detections carried out on simulated milk losses, i.e. when the time and characteristics of the emergence were known, were used to assess the sensitivity and timeliness of the method. Cluster detections carried out on the difference between observed and predicted milk productions, i.e. when there was no emergence to detect, were used to assess the specificity of the method.

### Factors Influencing Cluster Detection

#### Random noise

In order to mimic a predicted milk production that would have been totally unbiased but with various levels of unexplained variation, for each test-day that occurred in 2006, 3 random values were sampled from normal distributions with mean 0 and standard deviation 1, 3 and 5 respectively and added to the simulated milk production losses. The scan statistic was run on the 3 vectors of random values.

#### Disease characteristics

The association between disease characteristics and the ability of the scan statistic to detect their emergence was evaluated. Based on the results of the principal component analysis, 8 groups of diseases with similar characteristics were formed using the k-means algorithm. This allowed to carry out cluster detection on a subset of scenarios. Even though scenarios from different starting locations and dates can have similar features in terms of mean quantity of milk lost per cow per week and per zone, they may differ in terms of the number of cows recorded per week. Only scenarios starting from the Western starting location on the first of March were used at this step. The 8 scenarios fulfilling these selection criteria that were closest to the cluster centroids were selected.

#### Disease starting location and date

Cattle density is not homogeneous across France. Disease emergence simulations were started at both a high (West) and a low (North) cattle density location (Figure 3). Also, in France, milk recording is discontinued between mid-July and mid-August. Therefore, there would be less background data available to detect a disease emerging in early September than in early March. The associations between disease starting location and starting dates and cluster detection were tested. The scan statistic was run on 8 groups of scenarios with the same parameters as above but i) starting from the northern location ii) starting on the 1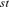 September.

#### Model performance

In order to determine the number of false alarms, cluster detection was carried out on the differences between predicted and observed milk production in 2006. Since there were only 6 farms with notified BTV cases during this year, all the alarms were considered to be false alarms. This was used as a measure of the method’s specificity.

## Results

### Expected Milk Production

**Figure 4:**
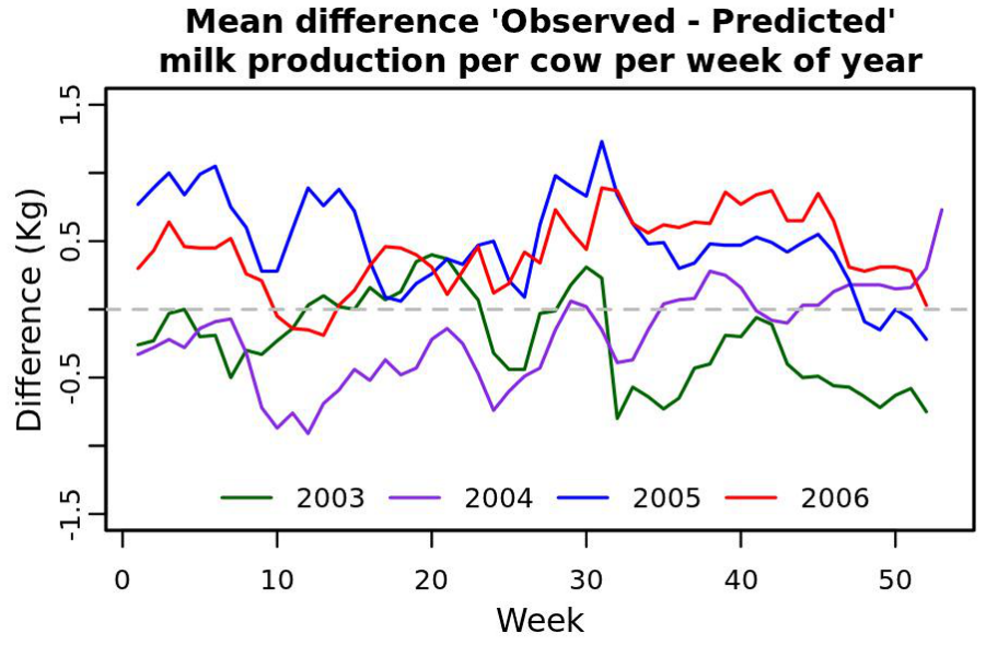
Difference between observed and predicted mean milk production per cow per week between 2003 and 2006. The years 2003 to 2005 were used for model fitting: the curves represent the mean of residuals per week. The year 2006 was used for disease simulation and detection.

A linear mixed model was fitted to monthly test-day milk productions collected between 2003 and 2005. The mean and standard deviation of the residuals were 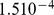 kg and 1.87 kg respectively. The model was then used to predict mean test-day milk productions per cow in 2006 and the differences between observed and predicted values were calculated for each test-day. The mean and standard deviation for the difference were 0.4 kg and 2.47 kg respectively. The mean difference between observed and predicted value per week of the year between 2003 and 2006 are presented in Figure 4. Although the model accounted for herd specific within year variation, there were systematic differences between recorded and predicted milk productions between each year. Except for weeks 10 to 14, observed milk production was always higher than predicted in 2006.

### Space-time Patterns of Disease Induced Milk Losses

**Figure 5:**
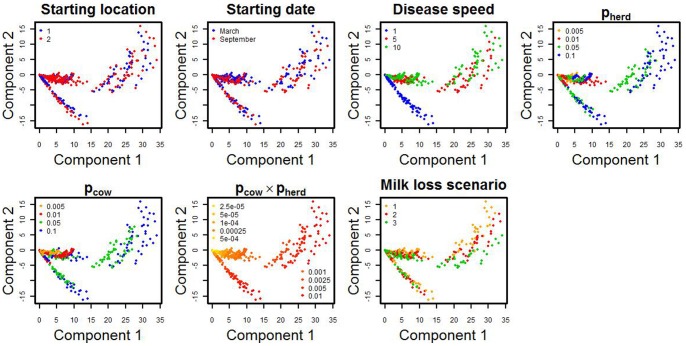
Association between emergence model parameters and the projection of each scenario on the 2 principal components identified in the principal component analysis. Each dot represents a scenario and each color represents a parameter level.

Each scenario was summarised by a vector of 64 values of mean production loss per cow for 8 geographical zones centred on the disease starting locations and for 8 weeks following disease emergence. The first 2 components of the principal component analysis explained 82.9% and 9.7% of the variability respectively. These 2 components were used to describe all the scenarios. Figure 5 displays the input parameters used for each scenario and the scenarios’ projection on the 2 principal components. The associations between scenarios’ input parameters and projection on the principal components were assessed visually. The most visible association was between the first component and the product of the daily probabilities of infection in herds within municipality (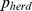) and in cows within herds (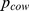). The only other parameter associated with the principal components was disease speed. Diseases progressing at 1 km/day were grouped on a single line while diseases progressing at 5 or 10 km/day were more scattered and could take similar values for the 2 principal components.

### Association between Random Noise and Cluster Detection

**Figure 6:**
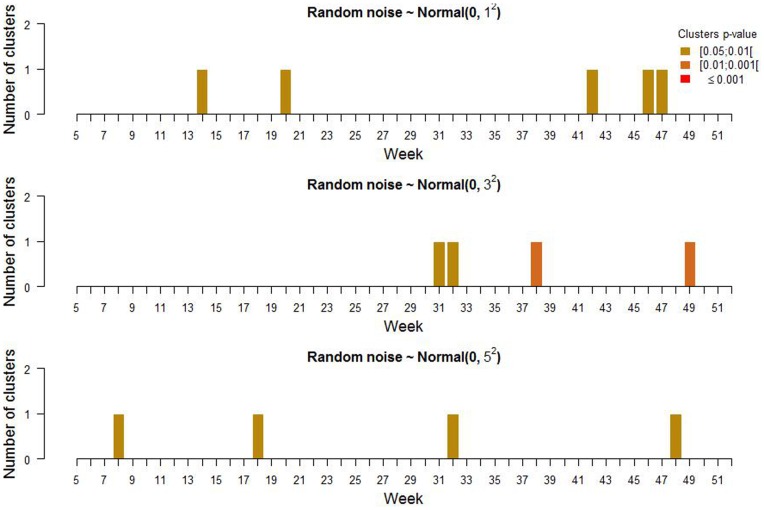
False alarms. Number of detected clusters of mean cow yield deviations for weeks 5 to 52.

Random values with mean 0 and standard deviation 1, 3 and 5 were assigned to each test-day that occurred in 2006. Cluster detection was carried out on the 3 vectors of random values separately. For each week under investigation, data for the 4 preceding weeks were also analysed with SaTScan™ as background information. Therefore, 47 out of 52 weeks were analysed per year. Clusters with p-value smaller than 0.05 were reported. The numbers of detected clusters per week for these 3 levels of random noise are presented in Figure 6. The maximum number of clusters detected in any week for the entire country was one. Of the 13 detected clusters, only 2 occurred in a week directly following another detection. Increasing the amount of noise did not change the number of detected clusters.

### Association between Disease Characteristics and Cluster Detection

**Figure 7:**
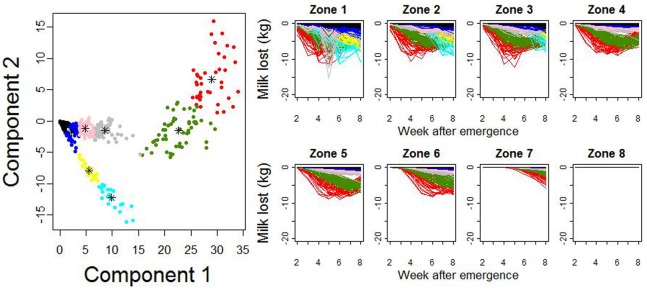
Groups generated using k-means algorithm from the scenarios 2 principal components. On the left-hand side plot, each dot represents a scenario and each color represents one of the 8 retained scenario groups. The plot on the right-hand side represents the mean quantity of milk lost per cow in each of 8 geographical area and during the first 8 weeks after emergence. The same colors are used on both sides.

**Figure 8:**
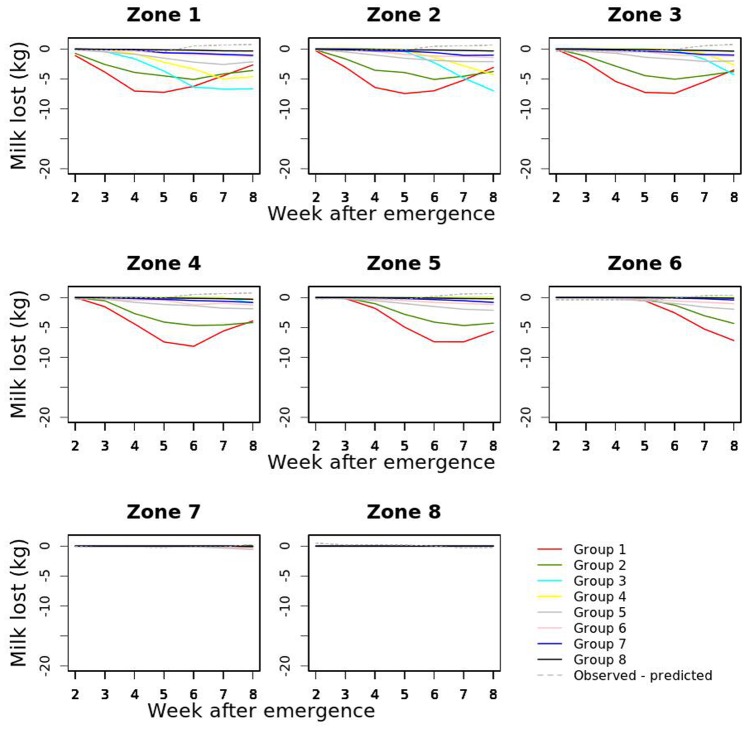
Comparison of the mean quantity of milk lost per cow in each of the 8 zones and during the first 8 weeks after emergence between the 8 selected scenarios and difference between milk production as observed and as predicted from the model results.

**Figure 9:**
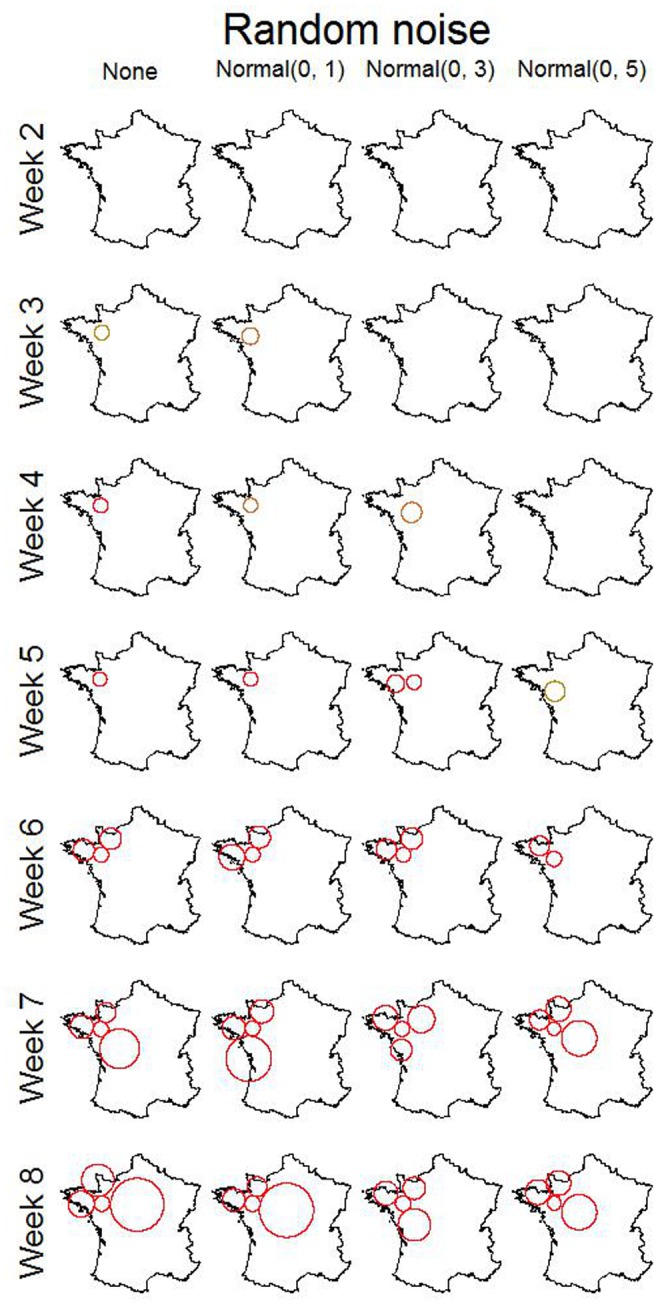
Detection of emergence induced milk losses by SaTScan for group 6 for weeks 2 to 8 after emergence. The scan statistic was performed on the simulated milk losses to which random noise was added.

The association between disease characteristics and the ability of the scan statistic to detect their emergence was evaluated. Cluster detection was carried out on the 8 scenarios selected from the groups formed using the k-means algorithm (Figure 7 and 8). The parameters used for the simulation of the selected scenarios are presented in Table 3. The intervals between disease emergence and disease detection for these scenarios are presented in Table 4. As an example, the clusters detected for group 6 are shown in Figure 9. Using the exact simulated milk losses as input, all diseases were detected within 3 weeks after their emergence. As could be expected, the time to first disease detection depended on the intensity of milk losses (See Figure 8). Adding random noise to the simulated milk losses did not have a consistent association with time to disease detection. It generally increased the time to detection, although it also lead to a detection earlier by 1 week in some cases where the random noise standard deviation was 1 or 3.

**Table 3:** Parameters used for the simulation of the 8 selected scenarios representing 8 disease groups.

Group	*S*	*P_herd_*	*P_cow_*	Milk loss scenario
1	5	0.1	0.1	Sc 2
2	5	0.05	0.05	Sc 1
3	1	0.1	0.1	Sc 3
4	1	0.05	0.05	Sc 2
5	10	0.01	0.1	Sc 3
6	10	0.005	0.1	Sc 3
7	5	0.005	0.05	Sc 3
8	5	0.01	0.005	Sc 3

The abbreviations used and the milk loss scenarios are presented in Tables 1 and 2.

**Table 4:** Number of weeks between disease emergence and cluster detection for groups of scenarios 1 to 8 for various levels of random noise added to the simulated milk losses.

Group	None	N(0, 1)	N(0, 3)	N(0, 5)
1	1	+1	+2	+3
2	1	+1	−1	+2
3	2	+1	+3	0
4	2	+3	+3	–
5	2	−1	0	0
6	2	−1	−1	0
7	2	0	+1	+2
8	3	−1	0	0

Cluster detections were performed using a scan statistic for groups of scenarios 1 to 8 on simulated milk losses as well as on simulated milk losses plus 4 levels of random noise. Random noise vectors were sampled from normal distributions with means 0 and standard deviations 1, 3 and 5. Disease emergence was simulated on week 9 (01/03/2006), starting from the Western location. The *None* column gives the week after emergence at which the first cluster was detected using the simulated milk losses without any added random noise as input. Other columns present the delay in weeks between cluster detection in the absence of noise and for the 3 simulated levels and random noise.

### Association between Disease Starting Location and Date and Cluster Detection

The number of weeks between disease emergence and cluster detection for the 8 groups for the 2 starting locations and the 2 starting dates are provided in Table 5. In all cases, clusters were detected at least equally fast after emergence at the starting location with the highest cattle density. For groups 1 and 3, clusters were detected one week later in the area with the lowest cattle density and for group 4, they were detected 3 weeks later. For the association between cluster detection and disease starting dates, in all cases, clusters were detected at least equally fast for diseases emerging in March. For groups 2, 6, 7 and 8, clusters were detected 1 week later when the emergence was simulated to start on the 1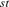 of September.

**Table 5:** Number of weeks between disease emergence and cluster detection for groups of scenarios 1 to 8 for 2 disease starting locations and 2 starting dates.

Group	West/March	North/March	West/Sept.
1	1	+1	0
2	1	0	+1
3	2	+1	0
4	2	+3	0
5	2	0	0
6	2	0	+1
7	2	0	+1
8	3	0	+1

The reference were diseases emerging at the western locations (High cattle density) on the 1st March and were compared to disease starting at the Northern location (Low cattle density) on the 1st March and diseases emerging at the Western location on the 1st September (Data collection interrupted in between mid-July and mid -August).

### Association between Model Performance and Cluster Detection

**Figure 10:**
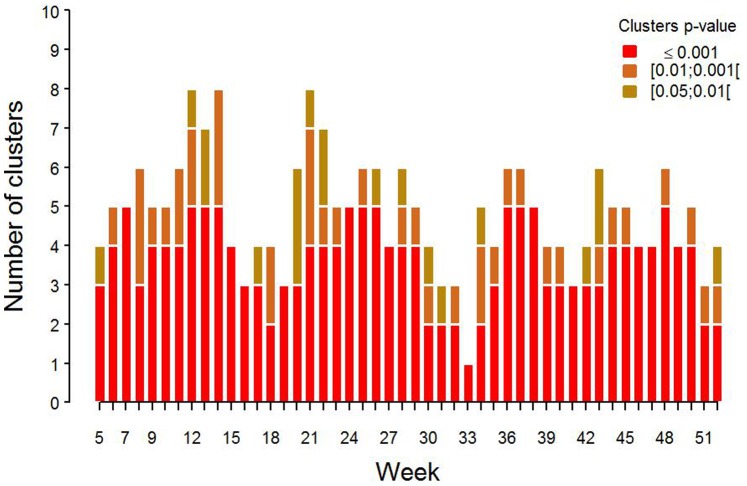
False alarms. Number of detected clusters for weeks 5 to 52 using the difference between observed milk production and milk production predicted from the model.

**Figure 11:**
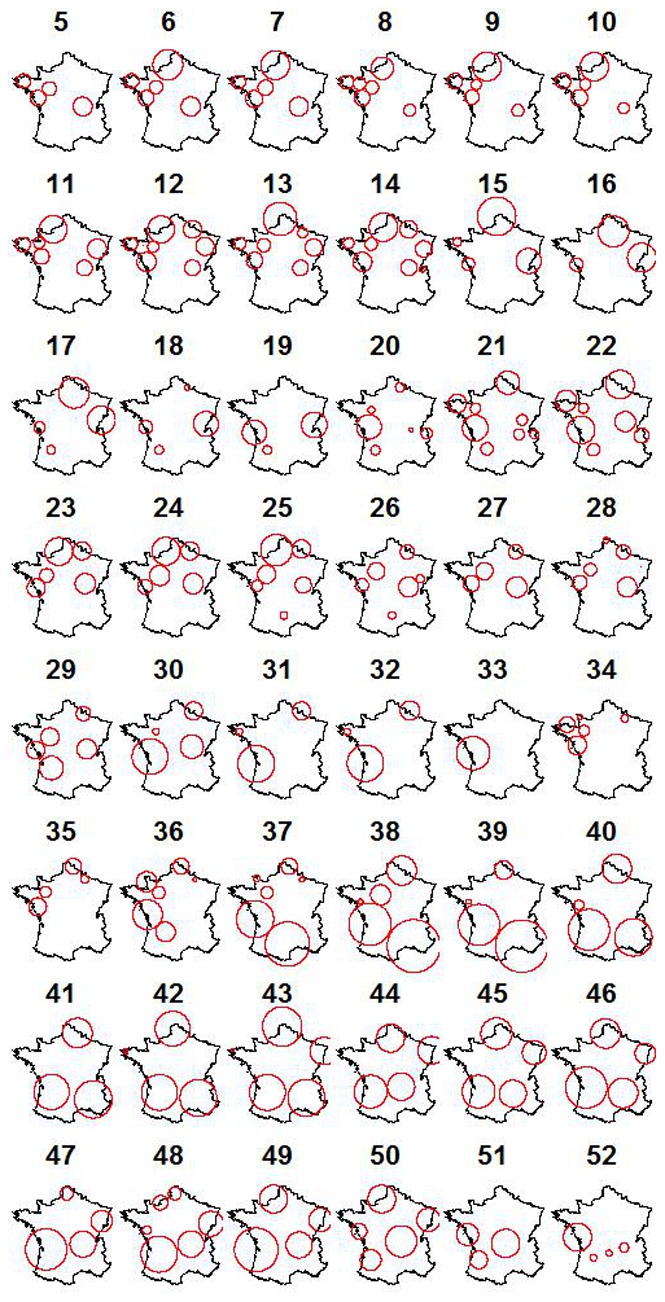
False alarms. Location of the detected clusters for weeks 5 to 52 using the difference between observed milk production and milk production predicted from the model. The number above each map is the week analysed.

The scan statistic was run on the difference between predicted and observed milk production in 2006. This allowed to quantify the number of false alarms returned by the scan statistic in the absence of an emergence (i.e. specificity) using predictions incorporating realistic levels of noise and bias. The mean (standard deviation) difference between observed and predicted milk production was of 0.4 (2.47) kg. There were between 1 and 5 clusters with p-value smaller or equal to 0.001 per week (Figure 10) and it was frequent for clusters to be present at the same location on consecutive weeks (Figure 11). Model bias is compared to disease induced milk losses for the scenarios representing the 8 groups defined above in Figure 8. Bias was of limited magnitude compared to decrease in milk losses caused by the simulated diseases.

## Discussion

When the disease to be detected is unknown, the sensitivity, timeliness and specificity of a disease detection method can only be evaluated for a range of putative spatio-temporal spread characteristics and effects on the monitored indicators. In the case of milk yield as an indicator of vector borne disease emergence, specificity was the main limitation of the detection method performance. Specificity depended mostly on the unbiasedness of the predicted indicator values. When the simulated milk losses were analysed with the scan statistic, the specificity was high, and, the sensitivity and timeliness of the method depended mostly on the extent of the effect of the emerging disease on the monitored indicator. In other surveillance systems or when using different indicators, other components could prevail. Simulation is a useful tool to explore the strengths and weaknesses of a detection method and to allocate resources towards the improvements of particular components. In the present case, efforts should be devoted to improving the prediction of the expected milk production in order to decrease the number of false alarms.

Simulation has been used several times to assess the determinants of detection in surveillance [19]. For example, Kulldorff *et al.* [20] used simulation to evaluate disease outbreak detection methods. They generated 134,977 benchmark datasets with a random number of cases of a hypothetical disease or syndrome. In the present study, with 576 nation wide datasets, it would have been intractable to run the scan statistic on all simulated scenarios. The chosen approach was to define families of scenarios based on space-time patterns of disease induced milk losses. This allowed to explore a subset of scenarios that differed substantially. The selected number of 8 groups of scenarios investigated further was a compromise between the need to have a sufficient variety of situations and the time required to carry out the analyses.

In the case of vector borne emerging diseases, the disease characteristics that accounted most for the pattern of milk losses were disease speed (denoted front-wave velocity in [15]) and ease of spread (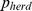 and 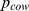) in the population. The effects of the disease on individual cow milk production was not important to predict the group in which a scenario would fall. It is possible that the model or the parameter space explored do not capture well the features of the diseases that could emerge. BTV was used as the reference around which simulation was based because it emerged recently in Europe and notification was mandatory in many countries. Thus this emergence has been well described in terms of spread and effects on production. Several studies using different assumptions and modelling techniques have been published on the spread of BTV [21]–[23]. The applicability of these models to vector borne diseases other than BTV is unknown but is likely to be limited in some cases. For example, since ticks cannot fly or be transported by wind, tick-borne diseases can be expected to spread much more slowly than culicoides-borne diseases. The model used to simulate disease spread was based on the minimum assumptions of circular progression, homogeneous probability of infection between farms within municipalities and homogeneous probability of infection between cows within farms. This set of assumptions was deemed sufficient to capture the main features of most vector borne diseases without being specific of a particular disease. There were limited data available to choose the parameters from. For bluetongue, Pioz *et al.* [15] estimated the speed of the epidemic front-wave to be of 5.6 km/day. Using a large milk recording dataset, Nusinovici *et al.* [16] estimated milk losses in infected herds between 2 and 3%. The input values used for the current work were consistent with this work. Santman-Berends *et al.* [24] gave lower estimates for the milk losses associated with BTV, but these were based on seroconversions with no associated clinical signs and were therefore not deemed representative of what happened during the start of the epidemic. The peak and duration of the effect were varied in order to see whether this would affect detection. For the values explored, these parameters explained very little of the variation in spatio-temporal patterns of milk losses. Other parameters were selected to reflect plausible values. With prospective disease surveillance, the event to detect is unknown. What is important is to use parameters that explore realistic scenarios. For example, using higher values for milk losses or ease of spread would have resulted in quicker detection, but would not have conveyed more information about the strengths and weaknesses of the method.

The scan statistic with a normal probability model was introduced in [12] for the detection of clusters of low birth weights in New York city. This method cannot be used because milk production undergoes seasonal variation that can be different between farms, which required a preliminary modelling step [25]. The main limitation of our method’s performance was the bias in predicted values. This was because there were predicted values that were systematically lower than the observed values and the differences were clustered in space as well as in time. This type of pattern is what would be expected if there were a vector borne disease to emerge. Therefore, the scan statistic behaved as expected and to improve its performance, the quality of the predictions must be improved. Systematic differences between observed and predicted values were even visible when predicting milk yields in the fitting dataset (2003 to 2005, see Figure 4). This was because milk production varies between years depending on factors such as for example climate, feed availability, feed price or quota extensions that are difficult to capture in a model. Our results do not invalidate the use of linear mixed models for the prediction of expected values. The incorporation of herd-time specific effects in the predictions accounted for breed, herd management and other unmeasured factors that are likely to be repeated between years. The implementation was straightforward using R and SaTScan™ in conjunction.

One way to overcome the problem of bias would be to decrease the threshold below which clusters are reported. In the present work, for clarity of presentation, all clusters with p-values smaller than 0.05 were reported. However, it would be possible to set a p-value, or a log likelihood ratio value, below which clusters would be considered to indicate disease. This value would be chosen so as to have less than a certain number of false positives throughout the year. This would decrease the power of the surveillance system but would ensure a manageable number of false positives. This will be the next step of our work.

Different choices could have been made regarding the level of data aggregation both in time and space. Milk recording data were available at monthly intervals for all the cows of a herd, but in a given area, there would be herds tested every day. Herd locations were available at the municipality level. One approach could have been to work with times series of data aggregated at some level, such as week/département as was done in [26]. Using time-series would have presented the disadvantage that the level of spatial aggregation has to be chosen *a priori*. Using large areas for spatial aggregation could delay detection by diluting the signal associated with emergence in random noise. Having small areas would result in having to analyse multiple time series with important random fluctuation in each series. The scan statistic does not impose constraints on the size of the cluster to be detected, except for the fact that a cluster cannot represent more than half of the population. Regarding the level of time aggregation, the week was chosen. The trade-off in this case was between the time required to undertake the analyses and the minimum detection delay between emergence and detection. A delay of 7 days between disease emergence and the triggering of an alarm seemed acceptable while the required computation time would be of less than 1 hour.

It is common in syndromic surveillance that what is to be detected is unknown. In order to test detection methods, a simple disease simulation model that produces a range of plausible disease effects on an indicator can be applied to real data in the absence of disease. It is then sufficient to run the detection method on a subset of simulated disease scenarios that are sufficiently different to determine the strengths and weaknesses of the methods. When monitoring continuous indicators, known factors of variation should be accounted for, especially when they are clustered in space and time. Linear mixed models can be used for this purpose. Milk yield from milk recording can be used for the detection of vector borne emerging diseases. The main limitation of this indicator was the difficulty to predict unbiased expected milk productions. Further work should be undertaken on improving the prediction of milk yield.
